# Antifungal Activity of Anionic Defense Peptides: Insight into the Action of *Galleria mellonella* Anionic Peptide 2

**DOI:** 10.3390/ijms21061912

**Published:** 2020-03-11

**Authors:** Aneta Sowa-Jasiłek, Agnieszka Zdybicka-Barabas, Sylwia Stączek, Bożena Pawlikowska-Pawlęga, Katarzyna Grygorczuk-Płaneta, Krzysztof Skrzypiec, Wiesław I. Gruszecki, Paweł Mak, Małgorzata Cytryńska

**Affiliations:** 1 Department of Immunobiology, Institute of Biological Sciences, Faculty of Biology and Biotechnology, Maria Curie-Skłodowska University, Akademicka 19 Street, 20-033 Lublin, Poland; a.sowajasilek@gmail.com (A.S.-J.); barabas@poczta.umcs.lublin.pl (A.Z.-B.); s.staczek@poczta.umcs.lublin.pl (S.S.); k-grygorczuk@wp.pl (K.G.-P.); 2 Department of Functional Anatomy and Cytobiology, Institute of Biological Sciences, Faculty of Biology and Biotechnology, Maria Curie-Skłodowska University, Akademicka 19 Street, 20-033 Lublin, Poland; bozena.pawlikowska-pawlega@poczta.umcs.lublin.pl; 3 Analytical Laboratory, Faculty of Chemistry, Maria Curie-Skłodowska University, M.C. Skłodowska Square 5, 20-031 Lublin, Poland; krzysztof.skrzypiec@poczta.umcs.lublin.pl; 4 Department of Biophysics, Institute of Physics, Faculty of Mathematics, Physics and Informatics, Maria Curie-Skłodowska University, M.C. Skłodowska Square 1, 20-031 Lublin, Poland; wieslaw.gruszecki@poczta.umcs.lublin.pl; 5 Department of Analytical Biochemistry, Faculty of Biochemistry, Biophysics and Biotechnology, Jagiellonian University, Gronostajowa 7 Street, 30-387 Krakow, Poland; pawel.mak@uj.edu.pl

**Keywords:** *Galleria mellonella*, *Candida albicans*, anionic defense peptide, antifungal peptide, atomic force microscopy, transmission electron microscopy, Fourier transform infrared spectroscopy

## Abstract

Anionic antimicrobial peptides constitute an integral component of animal innate immunity, however the mechanisms of their antifungal activity are still poorly understood. The action of a unique *Galleria mellonella* anionic peptide 2 (AP2) against fungal pathogen *Candida albicans* was examined using different microscopic techniques and Fourier transform infrared (FTIR) spectroscopy. Although the exposure to AP2 decreased the survival rate of *C. albicans* cells, the viability of protoplasts was not affected, suggesting an important role of the fungal cell wall in the peptide action. Atomic force microscopy showed that the AP2-treated cells became decorated with numerous small clods and exhibited increased adhesion forces. Intensified lomasome formation, vacuolization, and partial distortion of the cell wall was also observed. FTIR spectroscopy suggested AP2 interactions with the cell surface proteins, leading to destabilization of protein secondary structures. Regardless of the anionic character of the whole AP2 molecule, bioinformatics analyses revealed the presence of amphipathic α-helices with exposed positively charged lysine residues. High content of the α-helical structure was confirmed after deconvolution of the IR absorption spectrum and during circular dichroism measurements. Our results indicated that the antimicrobial properties of *G. mellonella* AP2 rely on the same general characteristics found in cationic defense peptides.

## 1. Introduction

Antimicrobial peptides (AMPs) or defense peptides constitute an essential component of innate immunity in invertebrates, vertebrates, and plants [1,2,3,4]. These peptides are usually small, highly cationic, and amphipathic molecules able to interact with lipid membranes. Amphipathicity is a key feature which determines peptide binding to a target cell membrane, whereas the cationic character is considered an important factor responsible for electrostatic attraction of the peptide by an overall negatively charged cell surface, typical for microorganisms. Many models describing interactions of AMPs with microbial cells assume that such electrostatic interaction between their negatively charged surface components and positively charged peptide molecules is an essential initial step of peptide binding. The negative charge of bacterial cells is determined by the presence of, e.g., lipopolysaccharide (LPS) and lipoteichoic acids (LTA), whereas the surface of fungal cells is often covered by anionic phosphomannoproteins. Moreover, differences in the phospholipid composition between the outer leaflet of prokaryotic and eukaryotic cell membranes resulting in anionic versus no charge ones are important for the selective toxicity of AMPs towards microbial cells. Interactions of AMPs with target cell membranes can result in a range of effects, including membrane depolarization, membrane permeabilization or lysis, and leakage of intracellular components, eventually causing cell death. Some cationic AMPs can traverse the cell membrane and target intracellular anionic constituents, such as DNA and RNA [5,6,7,8,9,10].

At present, almost 2330 of over 3140 defense peptides deposited in the Antimicrobial Peptide Database are of animal origin, of which more than 600 have been reported in invertebrates, including 310 in insects ([11,12], http://aps.unmc.edu/AP). Besides the large number of cationic AMPs isolated and characterized from different organisms, there are more than 100 examples of naturally occurring anionic defense peptides that exhibit high antimicrobial potential and are an integral and important part of the innate immune system. Such anionic peptides, although differing in the amino acid sequence and spatial conformation, have been described in insects, crustaceans, mollusks, amphibians, mammals, including humans, and plants. While many anionic AMPs are peptide fragments released by proteolytic cleavage of proteins playing a certain role in an organism, some anionic peptides are gene-encoded molecules [13,14]. The first anionic peptides were characterized in ovine pulmonary surfactant by Brogden et al. [15]. They were described as very short (7 amino-acid long), zinc-dependent peptides with activity against *Pasteurella haemolytica* and similar peptides were reported subsequently in cattle [16]. α-Helical maximin H5 and a defensin with pI 4.75 were isolated from the skin of the toad *Bombina maxima* and the tree frog *Polypedates puerensis*, respectively [17,18]. Defensin 2 with pI 4.44 with activity against *Escherichia coli* and *Staphylococcus aureus* was described in the tick *Amblyomma hebraeum* [19]. Anionic defensins were also detected in some insects, e.g., *Spodoptera littoralis* [20] and *Bombyx mori* [21]. In a vast majority of cases, animal anionic AMPs were examined against bacterial pathogens. The anionic AMPs of animal origin with antifungal activity are represented by human dermcidins [22,23], human platelet antimicrobial peptides [24], and histidine-rich PvHCt peptide generated from the C-terminus of hemocyanin in the panaeid shrimp [25,26]. The PvHCt peptide is active against filamentous fungi and exerts a fungicidal effect by inhibition of spore germination [25]. This 23-amino acid peptide (pI 6.16) adapted the amphipathic α-helical structure able to accumulate on the surface of *Fusarium oxysporum* hyphae. Insertion of the peptide into the fungal cell membrane probably caused local damage leading to disruption of membrane integrity, whereas the cell wall remained preserved [26].

A unique 7 kDa defense peptide with pI 4.79 named anionic peptide 2 (AP2) was reported in the greater wax moth *Galleria mellonella*, which is a common model insect species [27]. The amino acid sequence of *G. mellonella* AP2 was deposited in the UniProt/SwissProt database under accession number P85216 (ETESTPDYLKNIQQQLEEYTKNFNTQVQNAFDSDKIKSEVNNFIESLGKILNTEK KEAPK). This peptide is present constitutively in the hemolymph of naive *G. mellonella* larvae in a relatively high concentration of 12 µM and its synthesis is not induced by an immune challenge [28]; however, a decrease in its expression after injection of entomopathogenic bacteria *Bacillus thuringiensis* has been reported [29]. AP2 exhibits relatively low antibacterial activity against *Micrococcus luteus* and antifungal activity against yeasts belonging to the genus *Pichia*. However, AP2 acts synergistically with an antimicrobial protein of *G. mellonella* hemolymph, i.e., lysozyme, considerably enhancing its activity against Gram-negative bacteria and an opportunistic human pathogen, the yeast-like fungus *Candida albicans*, upon 24-h incubation [30,31]. Recently, Patiño-Márques et al. [32] have reported antiparasitic activity of a synthetic cationic 41-amino acid derivative of *G. mellonella* AP2 (amino acids from 20th to 60th of AP2: TKNFNTQVQNAFDSDKIKSEVNNFIESLGKILNTEKKEAPK) against *Leishmania panamensis*.

To provide more insight into the antifungal action of anionic AMPs, the effects of the *G. mellonella* AP2 used at physiological concentrations on the *C. albicans* survival rate, metabolic activity, cell surface topography, nanomechanical properties, and intracellular structures were examined. Moreover, Fourier transform infrared (FTIR) spectroscopy was applied for analysis of AP2 interaction with *C. albicans* cell surface components. In addition, FTIR and circular dichroism (CD) spectroscopy, as well as bioinformatics tools, were used for prediction of the properties and spatial structure of AP2 based on the peptide amino acid sequence. Considering the use of *G. mellonella* as a model organism in research on pathogenicity of different microorganisms, including *C. albicans* [33,34], the investigations on the AP2 antifungal action provide data on the role of this peptide in insect immune response against *C. albicans*, which are important for better understanding of the host-pathogen interactions in the *G. mellonella*-*C. albicans* system. In addition, this study contributes to an improved understanding of the antimicrobial properties of anionic peptides related to immunity.

## 2. Results

### 2.1. Survival Rate of C. albicans Treated with AP2

**Figure 1:**
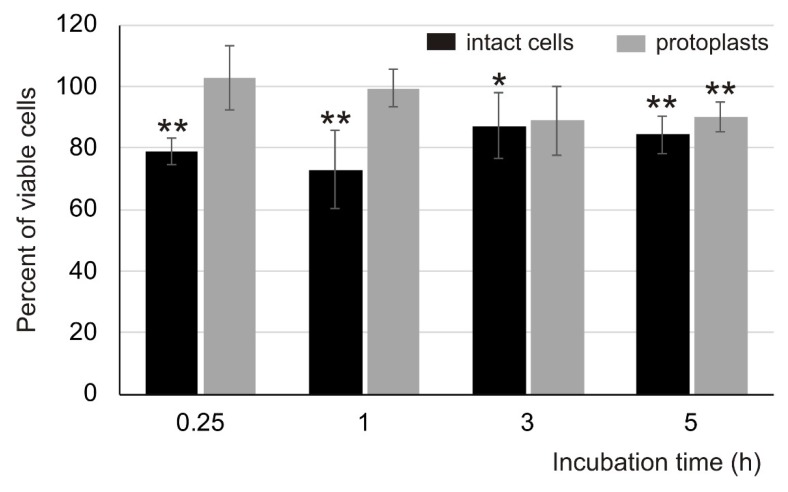
Survival rate of intact *C. albicans* cells and protoplasts treated with anionic peptide 2 (AP2). The log-phase intact cells or protoplasts (approximately 1 × 10^3^ colony-forming units (CFU)) were incubated without (control) or in the presence of 5 µM AP2 at 37 °C. Then, the cells were plated onto solid YPD medium and the grown colonies were counted. Statistical analysis was performed using One-way ANOVA (the compliance of the experimental data with the normal distribution and homogeneity of variance have been previously verified successfully). The results are presented as ±SD from three independent experiments. Statistical significance: * *p* < 0.05, ** *p* ≤ 0.01 (AP2-treated intact cells and protoplasts versus control intact cells and protoplasts, respectively). Survival of cells incubated without the peptide was considered as 100%.

The treatment of intact *C. albicans* cells with AP2 (5 µM) resulted in an approximately 23 and 28% decrease in the fungus survival rate after the 15 min and 1 h incubation, respectively. The survival rate after the 3 and 5 h incubation was calculated as approximately 90% (Figure 1). Interestingly, the increase in the AP2 concentration to 10 µM did not decrease the fungus survival further. In the presence of this elevated AP2 concentration, the survival rate was approximately 75, 88, and 84% after the 1, 3, and 5 h incubations, respectively. On the other hand, the 15 min and 1 h exposure to AP2 did not influence the survival rate of *C. albicans* protoplasts, i.e., cell wall-deprived cells (survival rate 102.99 and 99.37% after 15 min and 1 h incubation, respectively), whereas the longer incubation (3 and 5 h) resulted in an approximately 10% decrease in the protoplast survival rate (Figure 1). These results clearly indicated that, in contrast to the protoplasts, the intact *C. albicans* cells were more susceptible to the AP2 activity during the short-time incubation. They also suggested an important role of fungal cell wall components in the AP2 action.

### 2.2. Effect of AP2 on the Metabolic Activity of C. albicans Cells

**Figure 2:**
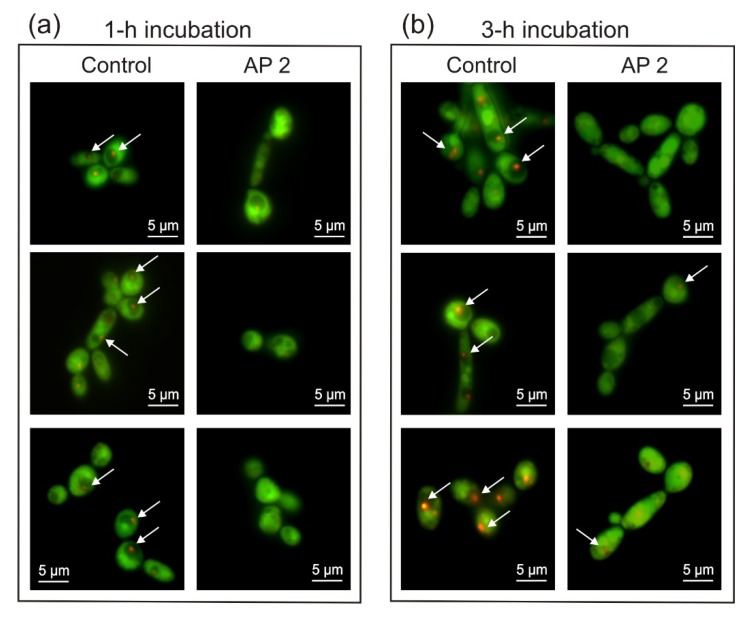
Effect of AP2 on *C. albicans* metabolic activity. The log-phase cells were incubated without (control) and in the presence of 5 µM AP2 at 37 °C for 1 h (**a**) and 3 h (**b**). The cells were then stained with FUN1 fluorescent dye and imaged using a laser scanning confocal microscope LSM 5 PASCAL. Metabolically active cells contain intravacuolar red-orange structures (indicated by white arrows). Representative images are presented.

The decrease in the survival rate of *C. albicans* upon the treatment with AP2 was correlated with a significant decrease in the metabolic activity of fungal cells estimated on the basis of formation of red-orange structures in vacuoles after FUN1 staining (Figure 2). The cells exposed to AP2 for 1 and 3 h exhibited approximately 37 and 60% lower metabolic activity in comparison with the activity of the control cells, respectively (Table 1). However, as demonstrated above in the survival rate analysis, most of the cells after the 3 h incubation with AP2 were still viable (Figure 1). This suggests that the AP2 action may cause temporary “weakening” of the cells, which probably facilitates the antifungal action of other immune factors.

The cells were incubated without (control) or in the presence of 5 µM AP2 at 37 °C. The cells were stained using Live/Dead Yeast Viability Kit and after imaging in a laser scanning confocal microscope, the percent of metabolically active cells was determined as described in Materials and Methods. Statistical analysis was performed using Kruskal-Wallis test. The data were presented as the means ± standard deviation (±SD) from three independent experiments.

### 2.3. Effect of AP2 on C. albicans Cell Surface Topography and Nanomechanical Properties

**Figure 3:**
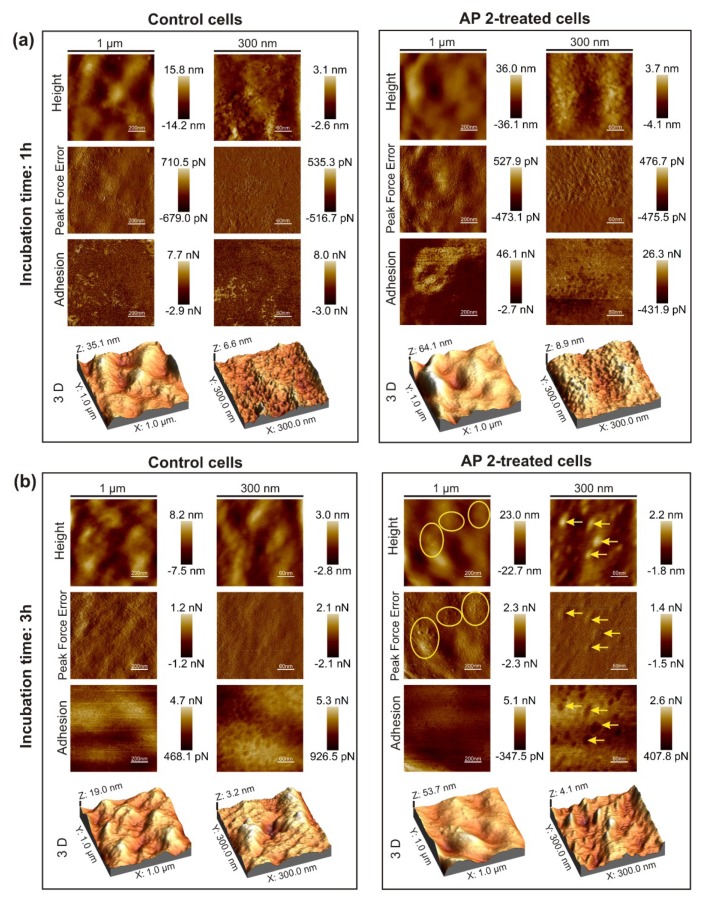
Effect of AP2 on *C. albicans* cell surface topography. The log-phase cells were incubated without (control) or in the presence of 5 µM AP2 at 37 °C for 1 h (**a**) and 3 h (**b**). The cells were then subjected to imaging by AFM. The height, “peak force error”, adhesion, and three-dimensional (3D) images are presented. The imaged areas of 1 µm × 1 µm (left panels) and 300 nm × 300 nm (right panels) are demonstrated. The yellow ellipses and yellow arrows indicate regions decorated with small clods and the clods, respectively.

The atomic force microscopy (AFM) analysis revealed alterations in the surface nanomechanical parameters of *C. albicans* cells exposed to AP2 for 1 and 3 h (Table 2). The most dramatic change was observed in cells after the 1 h incubation, for which adhesion forces increased approximately 17-fold and Young modulus increased 1.8-fold in comparison with the control cells. The cells exposed to AP2 for 3 h exhibited an approximately 1.6-fold decrease in both these parameters. On the other hand, the roughness of the cell surface was not affected by the studied peptide. Nevertheless, the surface of fungal cells treated with AP2 for 3 h was decorated with numerous well discernible small clods, which clearly exhibited higher adhesion forces than the surrounding surface (Figure 3).

The cells were incubated without (control) or in the presence of 5 µM AP2 at 37 °C and then imaged using atomic force microscope. The details of measurements of the cell surface nanomechanical properties are described in Materials and Methods. Statistical analysis was performed using Kruskal-Wallis test. The data were presented as the means ± SD from three independent experiments. 

### 2.4. Ultrastructure of C. albicans Cells Treated with AP2

**Figure 4:**
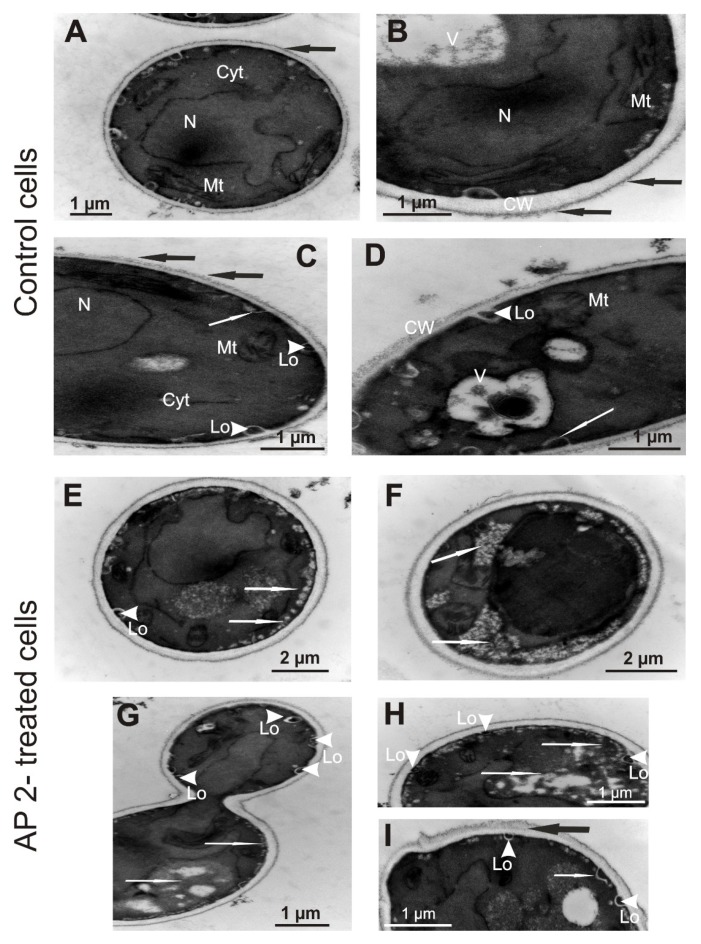
TEM images of *C. albicans* cells treated with AP2 for 1 h. The log-phase cells were incubated without (control; **A**–**D**) or with 5 µM AP2 (**E**–**I**) at 37 °C for 1 h. Then, the cells were imaged by TEM. **A**—control cell with well discernible cytoplasm (Cyt), nucleus (N), mitochondria (Mt), cell wall, and membrane. Note the presence of short, evenly distributed fibrils (black arrow) attached to the cell wall. **B**—enlargement of a fragment of the cell presenting cell wall (CW) with fibrils (black arrows), big vacuole (V), nucleus (N). **C**,**D**—fragments of the cells from the control culture with well visible fibrils (black arrows), big vacuoles (V), and other organelles. Cell membrane invaginations (white arrows) and lomasomes (Lo, white arrowheads) are visible. **E**,**F**—cells from the culture incubated with AP2 in which the presence of many small vacuoles located in the peripheral part of the cell or inside the cell are visible (white arrows). **G**,**H**—cells with noticeable vacuolated regions in the cytoplasm, tiny vacuoles close to cell membrane (white arrows), and lomasomes (Lo, white arrowheads). **I**—cell with cell membrane invagination (white arrow), lomasomes (Lo, white arrowheads), and some portion of the cell wall with distorted structure (black arrow).

**Figure 5:**
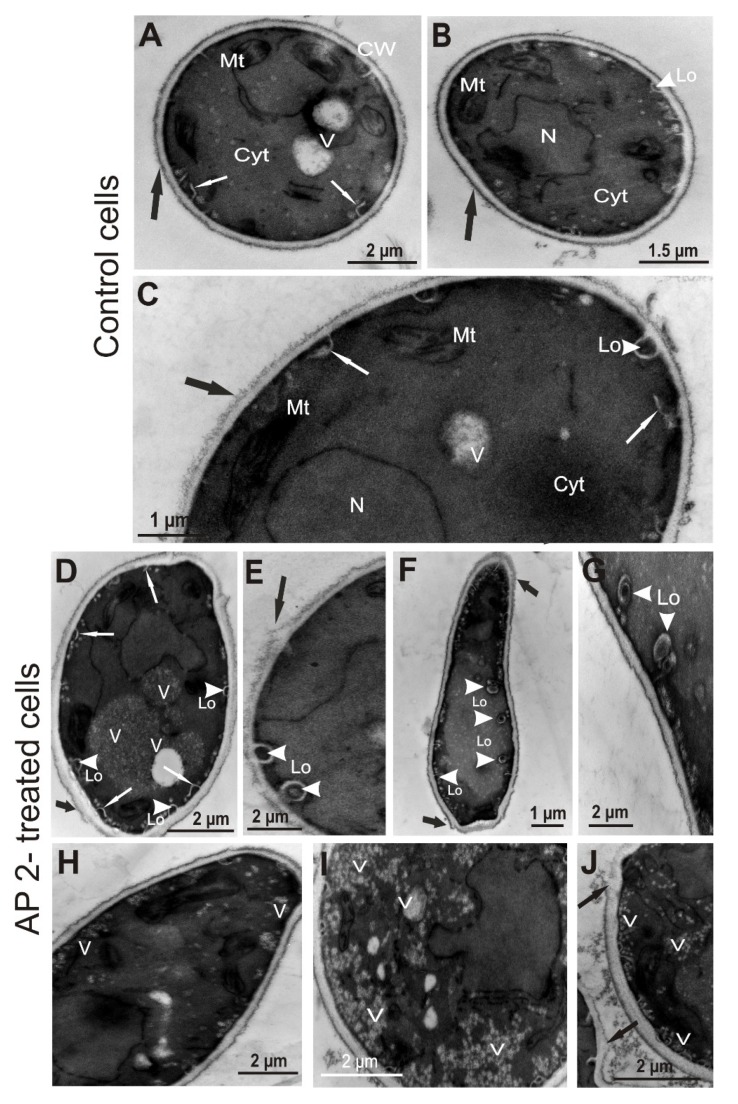
TEM images of *C. albicans* cells treated with AP2 for 3 h. The log-phase cells were incubated without (control; **A**–**C**) or with 5 µM AP2 (**D**–**J**) at 37 °C for 3 h. Then, the cells were imaged by TEM. **A**,**B**—control cells with well discernible cytoplasm (Cyt), nucleus (N), mitochondria (Mt), vacuoles (V), cell wall (CW) and short, evenly distributed fibrils (black arrows) attached to the cell wall. In (A) invaginations of the cell membrane are indicated by white arrows. In (B) a lomasome (Lo) is marked with white arrowhead. **C**—a portion of the control cell showing clearly the presence of cell wall fibrils (black arrow), a lomasome (Lo, white arrowhead), and cell membrane invaginations (white arrows). **D**,**E**—AP2-treated cells with some distortion of the cell wall (black arrows), many membrane invaginations (white arrows) and lomasomes (Lo, white arrowheads), and big vacuoles (V). **F**,**G**—a cell and its enlarged view with well discernible lomasomes in the form of vesicular bodies (Lo, white arrowheads) inside the cell. **H**–**J**—fragments of cells with vacuolated cytoplasm (V) and some distortion of the cell wall (black arrows).

The cells from the control cultures had a well discernible cell wall with outer brush-like and amorphous layers. The outer brush-like layer appeared to consist of short, evenly distributed oligomannan fibrils known to modify cell wall surWface proteins [35,36]. The cell membrane was attached to the innermost layer of the cell wall. In the cytoplasm of these cells, there were round-lobate nuclei, mitochondria, and big vacuoles. Characteristic invaginations of the cell membrane, as well as few lomasome-like structures, were visible, as well (Figure 4A–D and Figure 5A–C). When the *C. albicans* cells were exposed to AP2 for 1 and 3 h, some changes in the cell ultrastructure were observed. The presence of many small vacuoles located in the peripheral part of the cell or inside the cell was noted (Figure 4E–H and Figure 5I). In addition to the cell membrane invaginations detected in the control cells, many separated lomasomes in the form of vesicular bodies were also found near the cell membrane (Figure 4G–I and Figure 5E–G). In some cells, distortion of the amorphous layer of the cell wall was visible (Figure 5D–F,J). At the longer incubation time (3 h), more pronounced vacuolization and a greater number of lomasomes were noted (Figure 5D–J). In addition to these ultrastructural changes, the measurements of the cell wall thickness revealed a significant increase in this parameter in cells treated with AP2 for 3 h. The cell wall thickness was calculated as 31.63 nm (±5.37) and 48.09 nm (±9.41) for the control and the AP2-treated cells, respectively (*p* = 0.009).

### 2.5. FTIR Study of AP2 Interaction with C. albicans Cells

**Figure 6:**
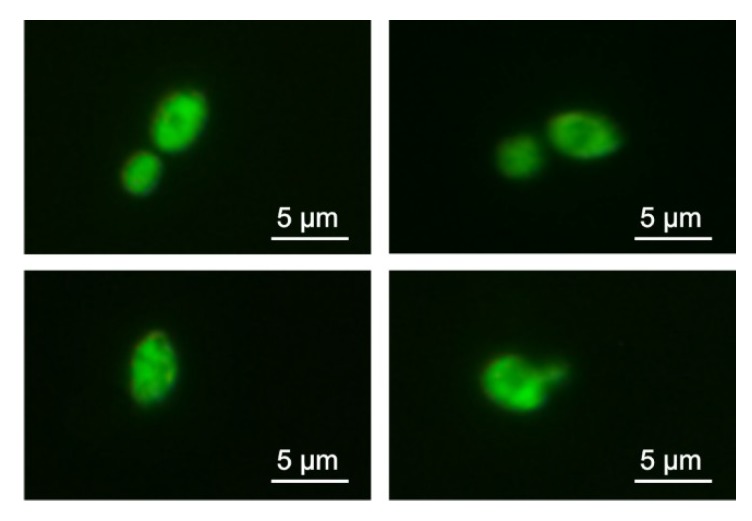
Binding of fluorescein isothiocyanate (FITC)-anionic peptide 2 to *C. albicans* cells. The cells were incubated in the presence of FITC-labeled AP2 (5 µM) at 37 °C for 15 min and then imaged by laser scanning confocal microscopy.

The possibility of AP2 interaction with *C. albicans* cells was first examined using the fluorescein isothiocyanate (FITC)-labeled peptide. The cells incubated in the presence of FITC-labeled AP2 exhibited green fluorescence when imaged in a laser scanning confocal microscope, indicating that the peptide can bind to fungal cells despite its anionic character (Figure 6).

**Figure 7:**
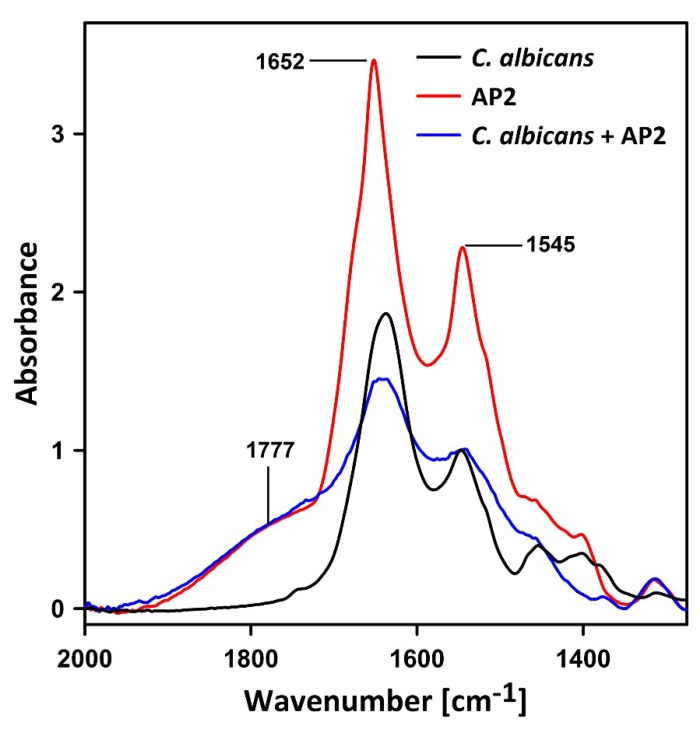
FTIR spectra of *C. albicans* cells treated with *G. mellonella* AP2. Infrared absorption spectra in the amide I and amide II region of anionic peptide 2 (AP2), *C. albicans*, and AP2-exposed *C. albicans* cells (marked). The spectra of *C. albicans* recorded before and after the incubation with AP2 were normalized at the maximum of the amide II band (1545 cm^−1^). The spectrum of pure AP2 was scaled to the same intensity as the spectrum of AP2-exposed *C. albicans* in the spectral region of 1777 cm^−1^, in which cells do not absorb IR radiation.

To shed more light in this aspect, FTIR analysis of the AP2-treated *C. albicans* cells was performed. Figure 7 presents the infrared absorption spectra of AP2, *C. albicans* cells, and *C. albicans* cells incubated with the examined peptide for 1 h. The spectral region comprises amide I (~1600–1700 cm^−1^) and amide II (~1500–1600 cm^−1^) bands, representing molecular vibrations in proteins. For comparison, the spectra recorded from *C. albicans* before and after the incubation with AP2 were normalized at the maximum of the amide II band (at 1545 cm^−1^). The spectrum of pure AP2 was scaled to the same intensity as the spectrum of *C. albicans* exposed to AP2 in the spectral region of 1777 cm^−1^, in which the cells do not absorb IR radiation. It is very likely that the relatively intensive and broad band in the AP2 spectrum centered at 1777 cm^−1^ originates from carbonyl groups exposed to a strong local electric field in the anionic peptide. As can be seen from the comparison of the spectra, the exposure of *C. albicans* to AP2 influenced strongly the shape of the amide I band, representing both the secondary structure and molecular organization of proteins. The most pronounced effect was associated with the decrease in the oscillator strength of C=O vibrations, manifested by a dramatic decrease in the amide I band intensity. Most probably, such an effect resulted from peptide-protein interactions of AP2 with the endogenous components of the cell surface and can be interpreted in terms of breakage of hydrogen bonds involved in stabilization of secondary protein structures. It is very likely that such effects are directly responsible for the activity of anionic peptide 2 towards the *C. albicans* cells. Spectral components in the amide I band origin from the shifts of the band representing C=O vibrations involved in the formation of hydrogen bonds stabilizing secondary structures of a protein. Position of such components on the energy scale and their relative intensity depend on excitonic interactions between neighboring hydrogen bonds. It is very likely that the local electric field generated by AP2, influences the excitonic interaction in proteins of *C. albicans* and possible protein-protein interaction (AP2 with proteins in cells) and that both those effects are collectively responsible for the relative broadening of the amide I and amide II bands, combined with the decreased intensities.

The FTIR measurements of AP2 alone suggested that the α-helix comprised approximately 50% of the AP2 molecule secondary structure. Figure S1 presents the results of deconvolution of the amide I spectral band of the peptide. The original IR absorption spectrum is presented along with the Gaussian components representing various secondary structure forms, assigned based on Tamm and Tatulian [37]. The comparison of the areas beneath each component demonstrated the following fractions with the forms of α-helix (48%), β-sheet (28%), turns and loops (15%), and antiparallel β-sheet (9%). These results were relatively well consistent with independent estimations from CD spectroscopy, which revealed the following conformations in native AP2 molecule: α-helix (42%), β-sheet (16%), turns and loops (27%) and random folds (15%) (Figure S2).

**Figure 8:**
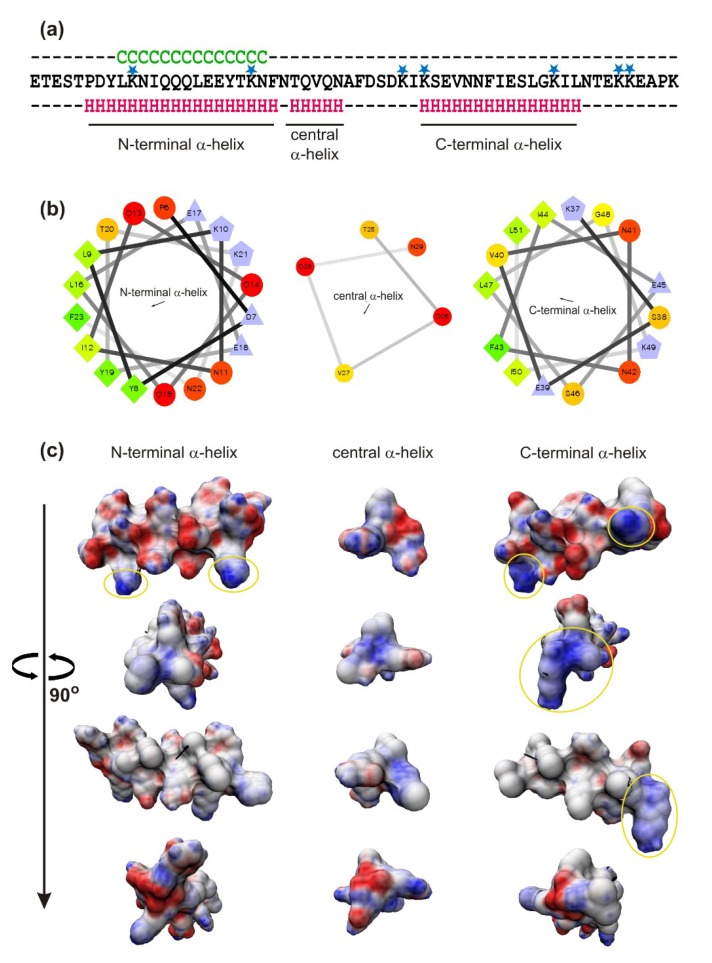
Prediction of α-helices and regions prone to aggregation (**a**), helical wheel projections presenting distribution of hydrophobic and hydrophilic amino acid residues (**b**), and distribution of electrostatic potential on the surface (**c**) of the α-helices predicted in *G. mellonella* anionic peptide 2. (a): the Jpred4 protein secondary structure prediction server available at http://www.compbio.dundee.ac.uk/jpred/ was used for prediction of α-helices (bottom line) and aggregation regions (upper line). Residues forming putative α-helices and involved in aggregation are marked H (pink letters) and C (green letters), respectively. Potentially exposed lysine residues (K) are indicated above the AP2 sequence by blue asterisks. (b): the hydrophilic, hydrophobic, potentially negatively charged, and potentially positively charged residues are represented by circles, diamonds, triangles, and pentagons, respectively. Hydrophilic uncharged and potentially charged residues are coded, respectively, red and light blue. Hydrophobic residues are coded yellow (zero hydrophobicity) to green (the most hydrophobic). The arrows inside the helical wheel projections indicate hydrophobic vectors (http://rzlab.ucr.edu/scripts/wheel/wheel.cgi). (c): the predicted N-terminal (left column), central (middle column), and C-terminal (right column) α-helices are presented as four pictures that represent rotations of 90 degrees around the z-axis (vertical). Positively and negatively charged areas are colored blue and red, respectively. The yellow ellipses encircle the most exposed positively charged regions. Non-polar regions are in gray. The electrostatic potential was analyzed using 3D-HM: The 3D Hydrophobic Moment Vector Calculator available as a web application on http://www.ibg.kit.edu/HM/.

The bioinformatics analysis (Jpred4 protein secondary structure prediction server) based on the AP2 amino acid sequence predicted three α-helical regions in the peptide molecule and one N-terminally placed fragment prone to aggregation (Figure 8A). Surprisingly, two of the putative α-helices, i.e., the N-terminal covering the 6th–23rd amino acids and C-terminal covering the 37th–51st amino acids, appeared to be amphipathic (Figure 8B) with positively charged lysine side chains well exposed on the surface of both the helices (colored blue in Figure 8C). Although amino acid sequence of *G. mellonella* AP2 has no obvious similarity to known polypeptides, attempts to predict the AP2 tertiary structure were undertaken using two different bioinformatics web-servers, SWISS-MODEL and Phyre2. The two models covering from the 14th to the 59th amino acids and from the 17th to the 51st amino acids of the AP2 sequence were obtained (Figures S3 and S4). While the modeling was performed on the basis of different templates, the presence of α-helices, as well as turns and loops in the peptide spatial structure, was indicated in both generated models.

## 3. Discussion

Research aimed at elucidation of the mechanisms of the antimicrobial action of anionic defense peptides are mainly focused on their activity toward bacteria. Studies on the antifungal action of anionic AMPs are less advanced and the modes of their activity are much less understood. Moreover, due to the great structural diversity of these molecules, results obtained for one peptide cannot be easily used for elucidation of the activity of others.

In the present study, the effects of the action of the unique anionic defense peptide, i.e., *G. mellonella* AP2, against *C. albicans* were examined using different microscopic techniques, whereas FTIR spectroscopy was applied for analysis of the peptide interactions with the fungal cells. In order to provide information helpful in elucidation of the interactions of the anionic peptide with the anionic surface of *C. albicans* cells, the properties and spatial structure of *G. mellonella* AP2 were also examined by FTIR and CD spectroscopy, as well as predicted using bioinformatics tools.

It is generally accepted that electrostatic attraction between AMPs and the negatively charged microbial cell surface is an important factor for their antimicrobial activity. The modification of *C. albicans* surface proteins by phosphomannosylation determines the generally negative charge of the *C. albicans* cell surface [38,39,40], which seems to exclude the electrostatic interaction with the anionic AP2. However, according to the bioinformatics analysis, the peptide molecule contains five cationic lysine residues with side chains exposed on the surface of the putative amphipathic α-helices and also three lysine residues on the C-terminus (Figure 8). This particular nine-amino acid C-terminal fragment may additionally expose the positive charge because beside the three lysine residues there are only two glutamic acid ones. Considering tertiary structure modeling of the AP2, the C-terminal α-helix may cover from 35th to 59th amino acids, including two lysine residues K55 and K56 (Figure S3). Taking together, these regions could considerably facilitate the attraction of the AP2 molecules to and their interaction with the negatively charged *C. albicans* cells. Designing AP2 analogs with lysine and/or glutamic acid residues replaced by other amino acids could help to elucidate the role of these charged residues in the AP2 interaction with *C. albicans* cell surface. A study on human anionic α-helical dermcidin-derived peptides, i.e., DCD-1L and DCD-1, revealed that their interactions with model lipid membranes were determined by the electrostatic attraction of cationic lysine residues of the peptide by negatively charged head groups of the membrane phospholipid [22,41]. The AP2 interactions with model lipid membranes have not been studied; nevertheless, the lack of susceptibility of *C. albicans* protoplasts demonstrated in our experiments indicated an essential role of the cell wall in the interaction of AP2 with the fungal cells.

The mannosylation of surface proteins mentioned above results in formation of a fibril layer comprising the outermost part of the *C. albicans* cell wall (demonstrated by TEM imaging in Figure 4 and Figure 5). Depending on environmental conditions, the oligomannans can form long, compact, and evenly distributed fibrils masking hydrophobic surface proteins, or they can form short and blunt fibrils that are unable to mask these proteins. These differences give rise to distinguishing hydrophobic and hydrophilic *C. albicans* cells grown at 30 and 37 °C, respectively [35,42]. The treatment of *C. albicans* cells with AP2 disturbed the outermost fibril layer characteristic of hydrophilic cells grown at 37 °C. Interestingly, the treatment of hydrophilic *C. albicans* cells with a reducing agent dithiothreitol or tunicamycin, an inhibitor of N-mannosylation of surface proteins [42,43], led to similar effects as those demonstrated after the AP2 treatment in this study. Given the clear amphipathicity of the predicted AP2 α-helices exposing lysine residues, the presence of the short cationic C-terminus, and the results of FTIR spectroscopy analysis demonstrating destabilization of the secondary structure of *C. albicans* surface proteins upon the AP2 treatment, it is possible that the unmasking of hydrophobic proteins by initial electrostatic attraction of the peptide facilitates further interaction of the peptide molecules with the yeast cell surface through hydrophobic interactions. Interestingly, the bioinformatics analysis of the AP2 sequence revealed a short region from the 9th to 22nd amino acid residue prone to aggregation. This feature might be responsible for the similar effects of the 5 µM and 10 µM concentrations of the peptide on *C. albicans* survival. On the other hand, it is well known that formation of oligomeric complexes by another anionic peptide, dermcidin DCD-1, is essential for its insertion into the bacterial membrane and antimicrobial activity [22,44]. However, further studies are needed to resolve the question whether AP2 molecules can form oligomers/aggregates between each other or with other peptides/polypeptides and in what conditions.

The *C. albicans* cells exposed to AP2 exhibited increased vacuolization, higher amount of lomasome-like structures, thicker cell wall and changes in the nanomechanical properties of the cell surface (especially an increase in adhesive forces). Lomasomes are intracellular structures located between the inner cell wall and the cell membrane. In *C. albicans* they appear as small vesicular bodies, large vesicular bodies, or a mass of coalescent vesicular body material [45]. These structures probably originate from the endoplasmic reticulum near the plasmalemma (called plasmalemmasomes), migrate outwards, and eventually fuse with the cell membrane. Interestingly, involvement of the lomasomes in transport of material required for cell wall formation in *C. albicans* has been proposed [46]. One can postulate that the exposure of the *C. albicans* cells to AP2 induced changes in the cell wall organization, which finally resulted in an increased cell wall thickness and were reflected by a transient but dramatic increase in the adhesion forces of the cell surface. It is worth mentioning here that a loss of adhesion force may imply that the pathogen cells are less able to adhere to surfaces. Changes in adhesion forces could contribute to a host survival, as well as yeast survival in the host, by altering the adhesion of the pathogen cells to host tissues. Our results also revealed a significant decrease in the *C. albicans* metabolic activity upon the exposure to AP2 for 1–3 h. The results of this study together with those reported previously [30] clearly indicated that such an effect of AP2 on *C. albicans* examined in vitro starts early and lasts up to 24 h. Temporary slowing down of metabolic activity may be a reaction of the fungus to AP2 sensing in the environment and may reflect an attempt to defend against the peptide, as it is generally accepted that cells with lower metabolic activity, e.g., stationary phase cells, are less sensitive to antimicrobial agents [47,48,49,50]. The protective response consisting of formation of daughter hyphae was observed in *F. oxysporum* exposed to the PvHCt anionic peptide [26]. On the other hand, the fact that there was no clear correlation between the decrease in the metabolic activity and the decrease in the survival rate in our study supports the idea that AP2 at physiological concentrations exhibits fungistatic activity against *C. albicans*. This activity was manifested by transient “weakening” of the cells, which probably facilitates the antifungal action of other *G. mellonella* immune factors during the early phase of antifungal immune response.

Summarizing, the fungal cell wall is important for anti-*C. albicans* activity of *G. mellonella* AP2. Binding of AP2 leads to decrease in metabolic activity of *C. albicans* cells, which is accompanied by alterations in cell surface topography and nanomechanical properties, as well as by intracellular changes. In the AP2 spatial structure amphipathic α-helices with exposed positively charged lysine residues were predicted by bioinformatics tools and high α-helical content was confirmed by FTIR and CD spectroscopic analyses of the peptide. Given that amphipathicity and exposure of the positive charge are considered two essential features for interactions of AMPs with microbial cells, the presence of these features in AP2 molecule indicates that they are also important for binding of this anionic peptide with anionic cell surface of *C. albicans*. It can be concluded that the antimicrobial properties of *G. mellonella* anionic peptide 2 rely on the same general characteristics found in cationic defense peptides. However, designing appropriate AP2 analogs would help explain the exact role of positively charged amino acids in the spatial structure, biophysical properties and antifungal activity of this unique anionic peptide.

## 4. Materials and Methods

### 4.1. Microorganisms

The yeast-like fungus *Candida albicans* (wild type; clinical isolate from the human mouth; Prof. Anna Kędzia, Department of Oral Microbiology, Medical University of Gdansk, Poland) was grown in YPD medium (1% yeast extract, 2% peptone, 2% dextrose) at 37 °C and stored on YPD slants solidified with 1.6% agar at 4 °C. For the experiments, the fungus was cultivated in ten-fold diluted YPD medium.

### 4.2. Preparation of C. albicans Protoplasts

The protoplasts were prepared using a mixture of *Trichoderma harzianum* lysing enzymes (Sigma-Aldrich, St. Louis, MO, USA) according to a protocol described in detail previously [51]. The log-phase *C. albicans* cells in YPD medium (OD_600_ = 1) were centrifuged (4000× *g*; 10 min; 4 °C) and the pellet was suspended in 0.1 M potassium phosphate pH 6. After the centrifugation, the cells were suspended in the same buffer supplemented with 2 M sorbitol and incubated in the presence of the lysing enzymes (final concentration 20 mg/mL) for 2 h at 28 °C with shaking. After the incubation, the cells were centrifuged as above, the pellet was washed with 0.1 M potassium phosphate pH 6 supplemented with 1 M sorbitol, and eventually the protoplasts were suspended in the same buffer. The number of protoplasts was evaluated by enumeration of colony-forming units (CFUs) after plating appropriate serial dilutions on YPD agar plates.

### 4.3. Purification and Fluorescent Labeling of Anionic Peptide 2

AP2 was purified from the methanolic extract of *G. mellonella* immune hemolymph according to the modified procedure described in our previous work [30]. Briefly, the lipid-deprived freeze-dried hemolymph extract dissolved in 0.1% (*v*/*v*) trifluoroacetic acid (TFA) was subjected to high-pressure liquid chromatography (HPLC) using a Discovery Bio Wide Pore C18 4.6 mm × 250 mm column (Sigma-Aldrich, St. Louis, MO, USA). The separation was carried out using two buffers, A: 0.1% TFA (*v*/*v*) and B: 0.07% TFA, 80% acetonitrile (both *v*/*v*), a linear gradient from 30 to 70% of buffer B in 35 min and a 1 mL/min flow rate. The fraction containing AP2 (a single peak eluting at approximately 26–27 min) was collected, freeze dried, and purified to homogeneity by additional separation on the same column as above and a linear gradient from 49 to 52% of buffer B in 20 min. The AP2 fraction (a single peak eluting at approximately 13–17 min) was freeze-dried and stored at −80 °C until further assays. The homogeneity and identity of purified AP2 was confirmed by SDS-PAGE electrophoresis [52] and by N-terminal sequencing performed using an automatic protein sequencer (Procise 491, Applied Biosystems). The quantitation of the peptide solution was determined by amino acid analysis [53]. The peptide was dissolved in sterile water before use.

The fluorescent labeling of AP2 was performed using fluorescein isothiocyanate (FITC, isomer 1, Sigma, St. Louis, MO, USA). A portion of AP2 containing 6 nanomols of the peptide was dissolved in 200 μL of sodium borate pH 8.5, mixed with 100-fold molar excess of an FITC dimethylsulfoxide solution, and incubated for 20 h at room temperature in darkness. To quench the unreacted FITC, 50 μL of a 1 M glycine solution were added and the mixture was incubated again for 0.5 h in darkness at room temperature. The labeled peptide was desalted using gel filtration chromatography on a Superdex Peptide 10/300 GL column (GE Healthcare, Chicago, IL, USA). The separation was performed at a 0.7 mL/min flow rate in 10 mM ammonium acetate buffer pH 5.8 containing 30% (*v*/*v*) acetonitrile. The single peak containing FITC-AP2 was collected and freeze-dried. Examination of the labeled peptide by SDS-PAGE revealed a single yellow band at ca. 7 kDa, which was fluorescent in the UV-light. The peptide was dissolved in sterile water before use.

### 4.4. Yeast Viability Assay

A CFU counting assay was used for determination of the AP2 effect on the survival rate of *C. albicans* intact cells and protoplasts, essentially as described previously [30,51]. Briefly, the log-phase *C. albicans* intact cells in ten-fold diluted YPD medium or protoplasts in sorbitol-supplemented phosphate buffer (OD_600_ = 0.2; approximately 1 × 10^3^ CFU) (final volume 12 µL) were incubated without (control) or in the presence of AP2 (2 µL; final concentrations: 5 µM and 10 µM) at 37 °C for 15 min, 1, 3, and 5 h. Sterile water (2 µL) was added to the control samples instead of the peptide solution. After the incubation, the suspensions were serially diluted, the cells were plated onto solid YPD medium, and after 24 h incubation at 37 °C, the grown colonies were counted. The survival rate was expressed as a percentage of the control, which was assumed to be 100%. The assay was performed on three independent occasions, each time in triplicate for the control and AP2-exposed cells.

### 4.5. Metabolic Activity Assay

The metabolic activity of *C. albicans* was determined using a LIVE/DEAD Yeast Viability Kit (Invitrogen) as reported previously [30,54]. The log-phase *C. albicans* cells (as described above) were incubated without (control) and in the presence of 5 µM AP2 at 37 °C for 1 and 3 h. To the control samples 2 µL of sterile water were added instead of 2 µL of the peptide solution. Then, the cells were centrifuged (3000 × *g*, 10 min, 4 °C), washed twice with 10 mM Na-HEPES pH 7.2, and finally were suspended in sterile GH buffer (2% glucose, 10 mM Na-HEPES pH 7.2). A FUN1 fluorescent dye solution was added to the suspension and, after 30-min incubation at 30 °C in darkness, the cells were imaged using a laser scanning confocal microscope LSM 5 PASCAL (Carl Zeiss, Oberkochen, Germany) (excitation and emission wavelength: 480 nm and 513 nm, respectively). Cells containing intravacuolar red-orange structures were considered metabolically active. From each probe, cells in twenty randomly chosen images were counted. The number of metabolically active cells was expressed as a percentage of all the cells. The assay was performed in three independent experiments, in triplicate for each type of probe.

### 4.6. Anionic Peptide 2 Binding to C. albicans Cells

The procedure was carried out essentially as reported previously [55] with some modifications. The log-phase *C. albicans* cells in ten-fold diluted YPD medium (20 μL; OD_600_ = 0.2) were incubated with 5 µM FITC-labeled AP2 at 37 °C for 15 min. Then, the cells were centrifuged (5000× *g*, 10 min, 4 °C), washed twice with phosphate-buffered saline (PBS; 20 mM phosphate buffer pH 7.4, 0.9% NaCl), and finally suspended in 10 μL of 20 mM phosphate buffer pH 7.4. The cells were imaged using a laser scanning confocal microscope LSM 5 PASCAL (excitation and emission wavelength: 470 nm and 520 nm, respectively).

### 4.7. Atomic Force Microscopy (AFM) Imaging and Analysis of C. albicans Cells

The log-phase *C. albicans* cells in ten-fold diluted YPD medium (12 µl; OD_600_ = 0.2) were incubated in the presence of 5 µM AP2 at 37 °C for 1 and 3 h. To the control samples 2 µl of sterile water was added. After washing with pyrogen-free water, the cells were centrifuged (7000× *g*, 10 min, 4 °C) and finally suspended in 5 µL of pyrogen-free water. The suspensions were applied on mica disks and dried at 28 °C, and the cells were subjected to imaging using a Nanoscope V AFM (Veeco, Plainview, NY, USA; Analytical Laboratory, Faculty of Chemistry, UMCS, Lublin, Poland). The measurements were performed in the PeakForce QNM operation mode using a RTESPA-300 silicon probe with spring constant 20–80N/m (Bruker Nano Inc. Billerica, MA, USA). Three fields (1 µm × 1 µm and 300 nm × 300 nm) were imaged for each sample. The average root-mean square (RMS) roughness values, Young modulus, and adhesion forces were calculated from twenty 200 nm × 200 nm fields measured on each 1 µm × 1 µm image. The nanomechanical properties were analyzed with NanoScope Analysis software ver. 1.40 (Veeco). Three-dimensional (3D) images were obtained using WSxM software (Nanotec, Tres Cantos, Spain).

### 4.8. Transmission Electron Microscopy (TEM) Imaging of C. albicans Cells

The log-phase *C. albicans* cells in ten-fold diluted YPD medium (OD_600_ = 0.2) were incubated at 37 °C for 1 and 3 h without (control) or with AP2 (final concentration 5µM) as described above. Afterwards, the cells were fixed for 2 h at 4 °C using 4.0% glutaraldehyde in 0.1 M cacodylate buffer pH 7.2 with addition of 0.8 M sorbitol and 5.5 mM CaCl_2_. Then, the samples were rinsed with 0.1 M cacodylate buffer and further fixed with 1.5% potassium permanganate in water for 1.5 h at 4 °C. After rinsing several times with deionized water, the cells were contrasted in 1% uranyl acetate for 1 h and dehydrated with 30, 50, 70, 90, and 100% ethanol. Finally, the cells were embedded in LR White Resin, polymerized at 55 °C overnight, and cut with a diamond knife into ultrathin sections (65 nm). The sections were collected on cooper grids and stained with 2% uranyl acetate for 15 min followed by Reynolds reagent (lead nitrate and sodium citrate) for 10 min [56]. All sections were examined with a Zeiss EM 900 electron microscope (Carl Zeiss, Germany). Additionally, measurements of the *C. albicans* cell wall thickness were performed using AxioVision release 4.8.2 program and the results were calculated on the basis of 25 cell measurements from the control and AP2-treated cells.

### 4.9. FTIR and CD Spectroscopy Analyses

Infrared absorption spectra were recorded using a Fourier-transform infrared absorption spectrometer Nicolet iS50R (Thermo Scientific, Waltham, MA, USA) equipped with an attenuated total reflection set-up (ATR-FTIR). An internal reflection element (diamond prism) was used as an attenuated total reflection element.

The log-phase *C. albicans* cells in ten-fold diluted YPD medium (0.5ml, OD_600_ = 0.2) were incubated without (control) or in the presence of 5 µM AP2 at 37 °C for 1 h. To the control samples the equivalent volume of sterile water was added. Then, the cells were centrifuged (5000× *g*, 10 min, 4 °C), washed with 20 mM phosphate buffer pH 7.4, and eventually suspended in 10 µl of the same buffer supplemented with 5% D_2_O (*v*/*v*). Addition of a small fraction of D_2_O enabled to correct the IR absorption spectra in the amide I region for contribution of water [57]. The samples were deposited on the ATR crystal element, evaporated, and the absorption spectra were recorded. The spectra of the pure AP2 prepared in water and the buffer alone both supplemented with 5% D_2_O (*v*/*v*) were also recorded. During the measurements, continuous purging with argon was applied. Typically, 10 scans were collected, Fourier transformed, and averaged for each measurement. Absorption spectra at a resolution of one data point every 2 cm^−1^ were obtained in the region between 4000 and 400 cm^−1^ using a clean crystal as the background. Spectral analysis and Gaussian deconvolution was performed with Grams Al software from Thermo Fisher Scientific (version 9.1, Waltham, MA, USA).

Circular dichroism (CD) spectrum of the pure AP2 was recorded in 185–250 nm range on J-715 spectropolarimeter (Jasco, Tokyo, Japan). The obtained curve was averaged from three independent measurements performed at room temperature using 30 μM AP2 solution in 20 mM phosphate buffer pH 7.4. The amount of secondary structures was estimated by fitting the measured spectrum to the reference spectra of five standard native proteins.

### 4.10. Bioinformatics Analysis

The bioinformatics analysis was based on the AP2 amino acid sequence (ETESTPDYLKNIQQQLEEYTKNFNTQVQNAFDSDKIKSEVNNFIESLGKILNTEKKEAPK) deposited in the UniProt/SwissProt database under accession number P85216. Prediction of α-helices and regions prone to aggregation was performed using the Jpred4 protein secondary structure prediction server available at http://www.compbio.dundee.ac.uk/jpred/ [58] (accessed on 17 April 2018). The helical wheel projections presenting the distribution of hydrophobic and hydrophilic amino acid residues were obtained using a web application available at http://rzlab.ucr.edu/scripts/wheel/wheel.cgi (accessed on 24 April 2018). The distribution of the electrostatic potential on the surface of the predicted AP2 α-helices was analyzed using 3D-HM: The 3D Hydrophobic Moment Vector Calculator available as a web application on http://www.ibg.kit.edu/HM/ [59] (accessed on 24 April 2018). For AP2 tertiary structure modeling, the SWISS-MODEL web server (accessed on 04 February 2019) and Phyre2 web portal (accessed on 14 February 2019) were used. The model covering from the 14th to the 59th amino acids of the AP2 sequence was generated on a template of borealin (template number 2qfa.1.B) in a SWISS-MODEL workspace available at https://swissmodel.expasy.org/ [60,61,62,63,64]. The model covering from the 17th to the 51st amino acids of the AP2 sequence was generated on a template of *Helicobacter pylori* hypothetical protein HP0242 (SCOP ID: d2oufa1) using the Phyre2 web portal available as a web application on http://www.sbg.bio.ic.ac.uk/phyre2/html/page.cgi?id=index [65].

## Figures and Tables

**Table 1:** Metabolic activity of *C. albicans* after treatment with *G. mellonella* AP2.

Incubation Time	Metabolic Activity (%)	
	Control Cells	AP2-Treated Cells
1 h	89.35 (±4.58)	56.05 (±11.15) *
3 h	84.73 (±7.28)	33.74 (±9.45) *

* Statistical significance. The differences between the peptide-treated and control cells at the given time point are statistically significant (* *p* < 0.05).

**Table 2:** Nanomechanical properties of *C. albicans* cell surface after treatment with *G. mellonella* AP2.

Incubation Time	Tested Parameter	Control Cells	AP2-Treated Cells
1 h	RMS roughness (nm)	1.59 (±0.59)	1.76 (±1.6)
	Young modulus (GPa)	2.85 (±0.95)	4.65 (±1.32) *
	Adhesion forces (nN)	1.07 (±0.48)	18.59 (±7.34) *
3 h	RMS roughness (nm)	1.14 (±0.48)	1.11 (±0.76)
	Young modulus (GPa)	2.56 (±0.28)	1.27 (±0.33) *
	Adhesion forces (nN)	2.42 (±0.88)	1.12 (±0.55) *

* Statistical significance. The differences between the peptide-treated and control cells at the given time point are statistically significant (* *p* < 0.05).

## Supplementary Materials

Supplementary materials can be found at https://www.mdpi.com/1422-0067/21/6/1912/s1.

## Abbreviations

AFM	atomic force microscopy
AMP	antimicrobial peptide
CD	circular dichroism
CFU	colony forming unit
FITC	fluorescein isothiocyanate
FTIR	Fourier transform infrared
HPLC	high-pressure liquid chromatography
LPS	lipopolysaccharide
SDS-PAGE	sodium dodecyl sulfate-polyacrylamide electrophoresis
TEM	transmission electron microscopy
TFA	trifluoroacetic acid
YPD	yeast peptone dextrose
