# Supplementary material for: Antifungal Activity of Anionic Defense Peptides: Insight into the Action of Galleria mellonella Anionic Peptide 2
Source: Int J Mol Sci. 2020 Mar 11;21(6):1912. doi: 10.3390/ijms21061912 (PMC7139982; doi:10.3390/ijms21061912)
Supplement: Supplementary file 1 [file ijms-21-01912-s001.pdf]

Supplementary Materials

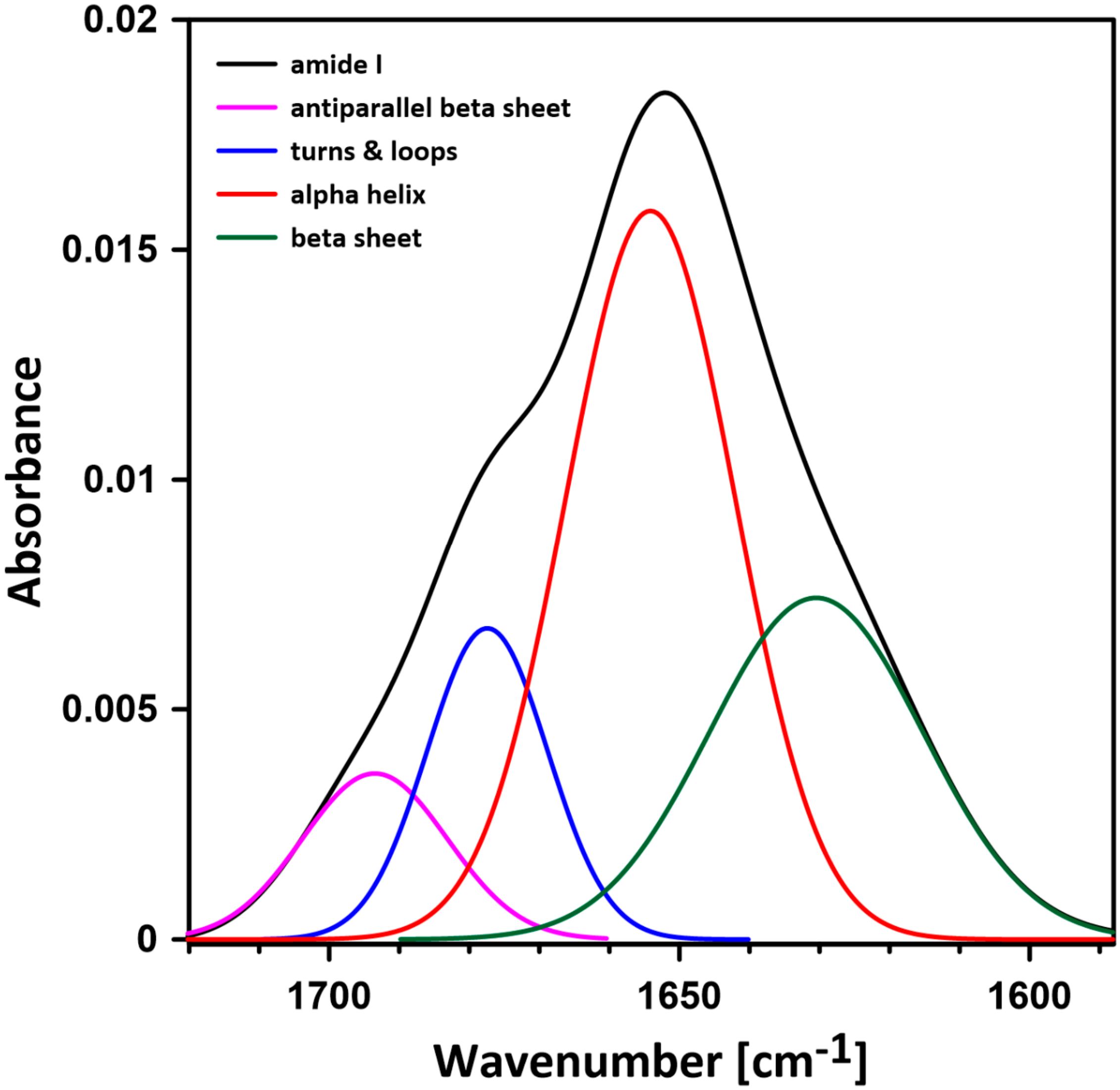

Figure S1. IR absorption spectrum of *G. mellonella* anionic peptide 2 in the amide I region. Overlapping components of the Gaussian deconvolution assigned to the secondary structure forms of the peptide are also presented (marked).

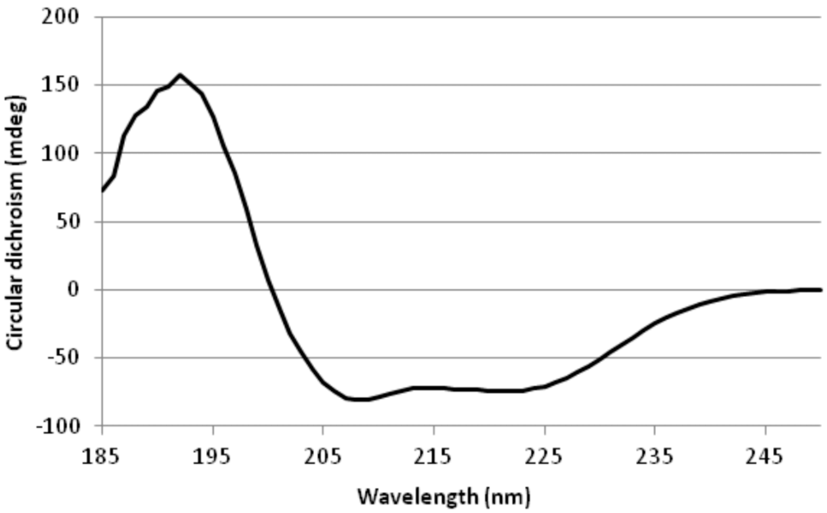

Figure S2. Circular dichroism spectrum of *G. mellonella* anionic peptide 2. The obtained curve was averaged from three independent measurements performed at room temperature using 30 μM AP2 solution in 20 mM phosphate buffer pH 7.4.

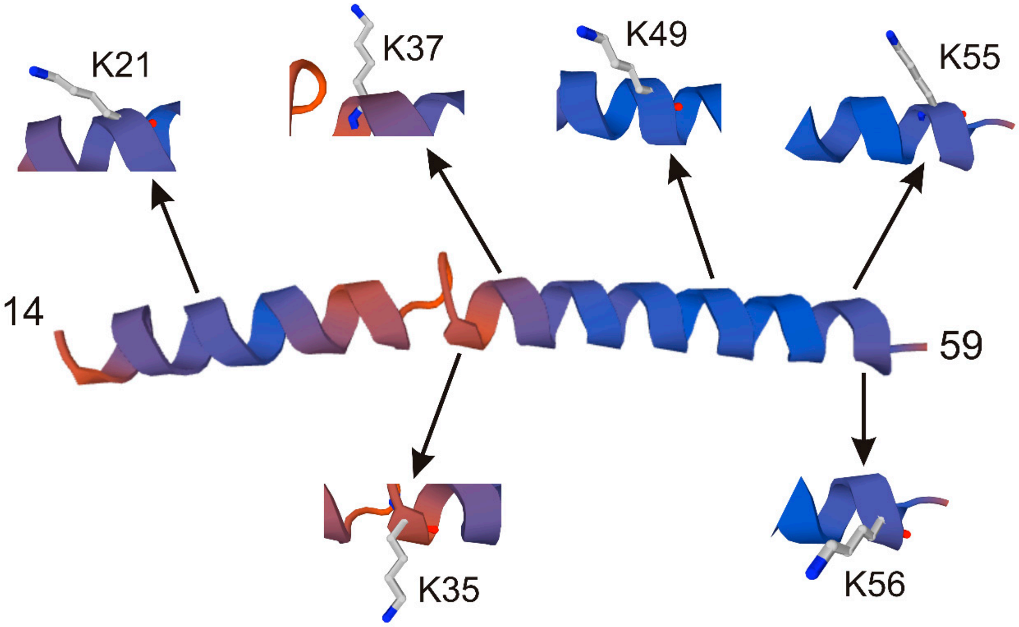

Figure S3. Tertiary structure modeling of *G. mellonella* anionic peptide 2. The model covering from the 14th to the 59th amino acids of the AP2 sequence was generated on a template of borealin using SWISS-MODEL Homology Modeling workspace available at https://swissmodel.expasy.org/. The per-residue model quality is indicated by colors (blue and red indicate the highest and the lowest quality, respectively). Exposed side chains of positively charged lysine residues are indicated.

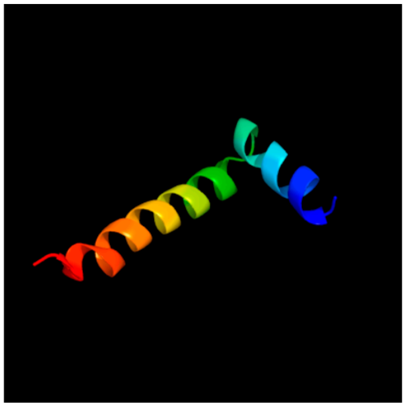

3D model of AP 2 (17-51aa)

Figure S4. Cartoon representation of *G. mellonella* anionic peptide 2 colored in rainbow from the N-terminus (blue) to the C-terminus (red). The model covering from the 17th to the 51st amino acids of the AP2 sequence was generated using the Phyre2 web portal available at http://www.sbg.bio.ic.ac.uk/phyre2/html/page.cgi?id=index. Confidence of the model: 63.2%.
